# Flexor Digitorum Accessorius Longus: Importance of Posterior Ankle Endoscopy

**DOI:** 10.1155/2015/823107

**Published:** 2015-04-28

**Authors:** Jorge Pablo Batista, Jorge Javier del Vecchio, Pau Golanó, Jordi Vega

**Affiliations:** ^1^Centro Artroscópico Jorge Batista, 2446 Pueyrredón Avenue, 1st Floor, 1119 Buenos Aires, Argentina; ^2^Favaloro Foundation, 461 Solis Street 1st floor, 1078 Buenos Aires, Argentina; ^3^Human and Embriology Unit, Department of Experimental Pathology and Therapeutics, University of Barcelona, Spain; ^4^Hospital Quirón Barcelona, Plaça d'Alfonso Comín, 08023 Barcelona, Spain

## Abstract

Endoscopy for the posterior region of the ankle through two portals is becoming more widespread for the treatment of a large number of conditions which used to be treated with open surgery years ago. The tendon of the *flexor hallucis longus* (FHL) travels along an osteofibrous tunnel between the posterolateral and posteromedial tubercles of the talus. Chronic inflammation of this tendon may lead to painful stenosing tenosynovitis. The aim of this report is to describe two cases depicting an accessory tendon which is an anatomical variation of the *flexor hallucis longus* in patients with posterior friction syndrome due to posterior ankle impingement and associated with a posteromedial osteochondral lesion of the talus. The anatomical variation (FDAL) described was a finding during an endoscopy of the posterior region of the ankle, and we have spared it by sectioning the superior flexor retinaculum only. The accessory flexor digitorum longus is an anatomical variation and should be taken into account when performing an arthroscopy of the posterior region of the ankle. We recommend this treatment on this type of injury although we admit this does not make a definite conclusion.

## 1. Introduction

Endoscopy for the posterior region of the ankle through two portals is becoming more widespread for the treatment of a large number of conditions which used to be treated with open surgery years ago [1–5].

No doubt this is mostly due to the significant contribution arthroscopic anatomy has meant for this particular region of the body [6, 7]. At present, it is also used for the treatment of prominent os trigonum, chronic synovitis of the* flexor hallucis longus* (FHL) [8], osteochondral lesions of the posterior region of the talus, loose bodies, subtalar arthrosis, and other less common conditions such as cystic-like bone tumors involving the talus and/or calcaneus and synovial chondromatosis.

The tendon of the* flexor hallucis longus* (FHL) travels along an osteofibrous tunnel between the posterolateral and posteromedial tubercles of the talus. Chronic inflammation of this tendon may lead to painful stenosing tenosynovitis, typically seen in dancers [8].

Activities including forced foot flexion such as running downhill or playing soccer are a predisposing factor for injuries involving the FHL. Moreover, os trigonum, cysts,* flexor digitorum accessorius longus* (FDAL), and talar dorsal exostosis may lead to tenosynovitis [8–10].

The aim of this report is to describe a clinical case depicting an accessory tendon which is an anatomical variation of the* flexor hallucis longus* in a patient with a posterior friction syndrome due to posterior ankle impingement. The discussion is based on the endoscopic diagnosis of the accessory FDL and the treatment established.

## 2. Case Report

A 34-year-old male (doing recreational sports) complained of a 6-month history of pain in the posterior region of his left ankle. Standing on tip toes triggered the symptoms.

**Figure 1:**
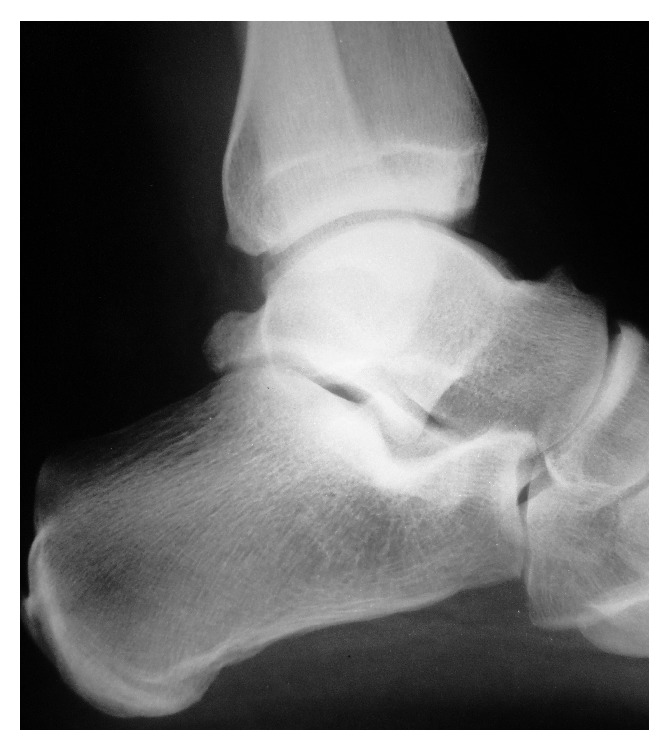
Lateral X-ray. Prominent posterior talar process.

**Figure 2:**
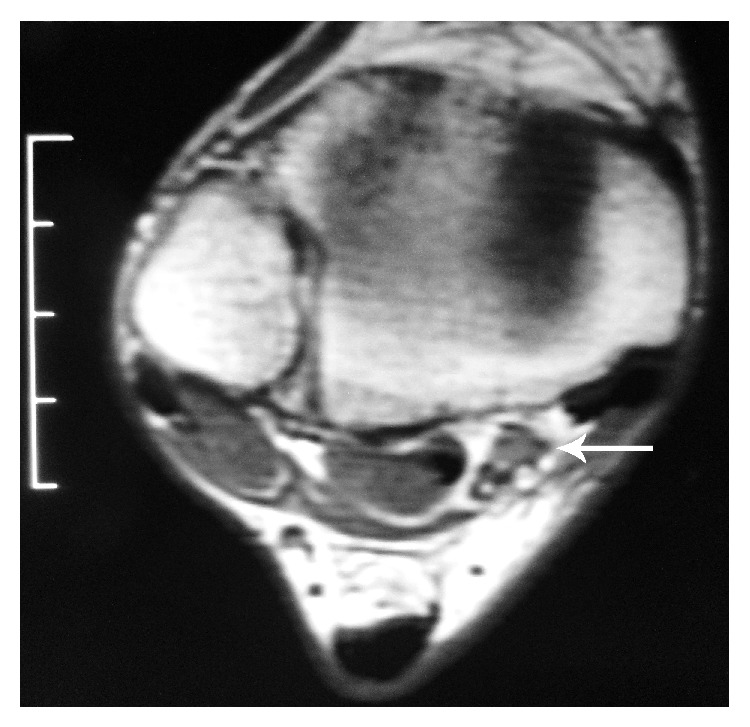
Accessory muscle (white arrow).

On physical examination, no pain was observed during either passive or active mobilization or hallux counter resistance (both plantar and dorsal flexion). The ankle hyperplantarflexion test was positive. There was also a mild limitation in movement of the lesser toes with the foot held in dorsiflexion. The lateral X-ray view evidenced the presence of a prominent posterior talar process (Figure 1). The MRI showed a muscular intensity image located close to the posterior tibial neurovascular bundle (Figure 2).

Since conservative treatment administered for 4 months (rest from sports activities, physiotherapy, and medical treatment) failed, and as symptoms remained, a posterior arthroscopy of the ankle was conducted in order to resect the posterior prominent area of the talus and then perform soft tissue debridement.

**Figure 3:**
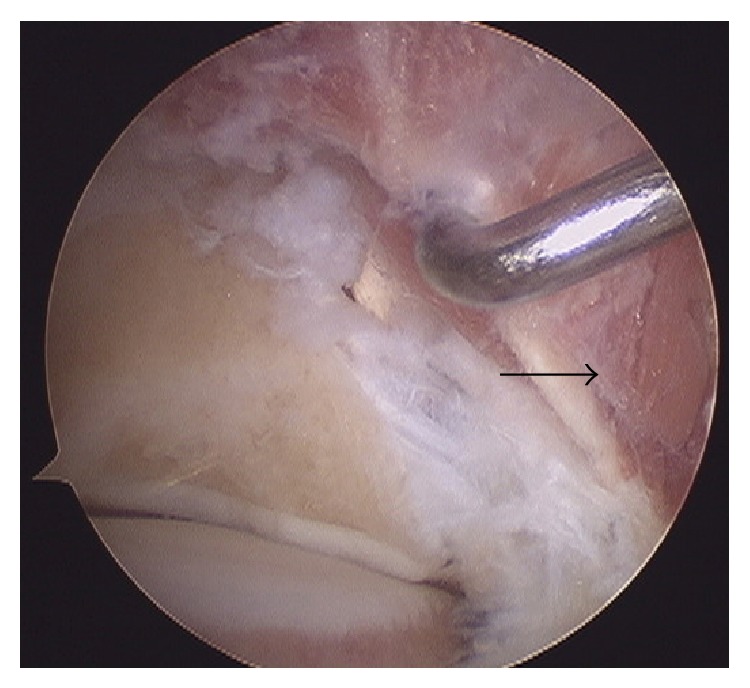
Accessory muscle belly (black arrow).

**Figure 4:**
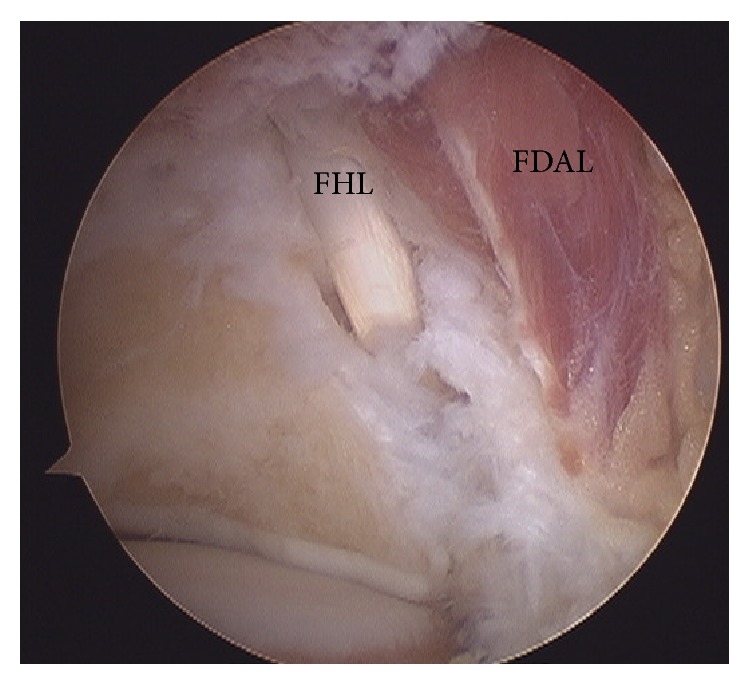
FHL and FDAL recognized.

During the procedure, a large accessory muscle belly was detected, and on dislodging it medially (Figure 3) the tendon of the FHL was identified. The muscle belly was considered an anatomical variation (FDAL) (Figure 4).

**Figure 5:**
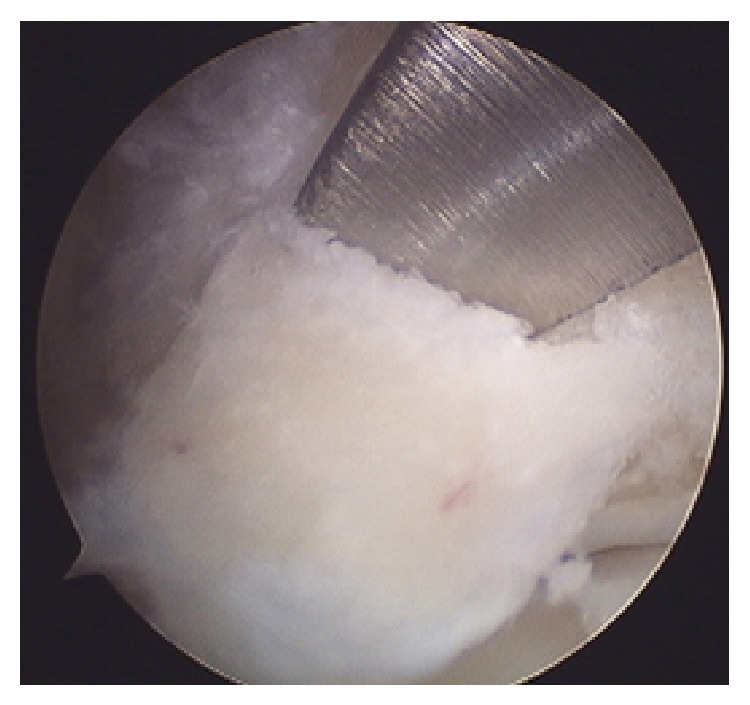
Resection of posterior talar process using a chisel.

**Figure 6:**
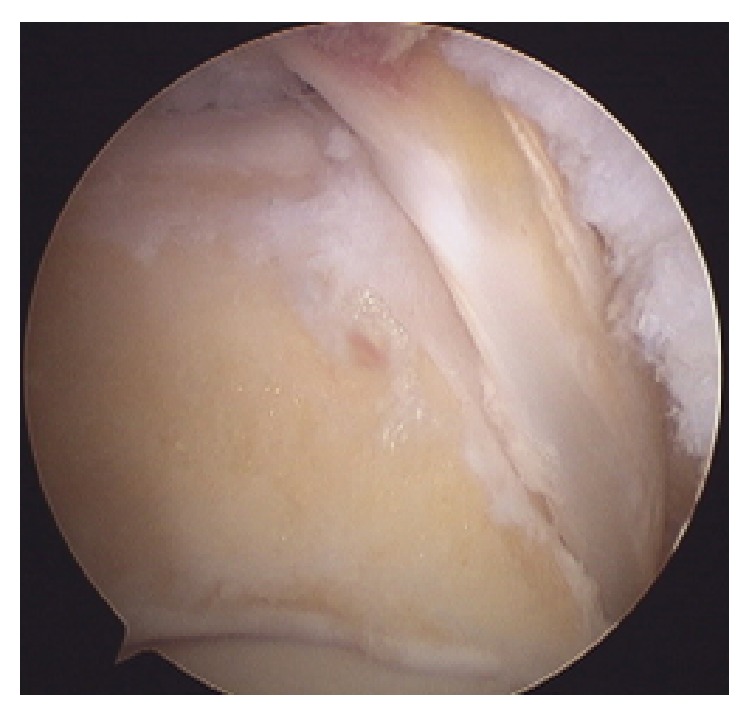
Complete resection.

The posterior talar process was resected by using both mechanical and motor instruments (Figures 5 and 6), and the superior flexor retinaculum was sectioned without involving the accessory muscle and the corresponding tendon (FDAL).

The ankle was bandaged, and active mobilization (flexion-extension) was started immediately after surgery. The patient went back to recreational sports within 3 months. At 19-month follow-up the patient did not have any related symptoms.

## 3. Discussion

This condition (FDAL) is more common among males although the difference between males and females is not significant. It is unilateral in most cases, although nine bilateral cases have been published [10–14].

According to Bowers et al. [15] the accessory muscle body of the FHL is the second leading cause of anatomical variation occurring in the ankle. The peroneus quartus is the most common, followed by other variations such as the accessory soleus muscle, the medial calcaneal tibial muscle, and the medial fibular-calcaneal muscle.

The accessory muscle body of the FHL may be an anatomical variation identified accidentally during a posterior arthroscopy of the ankle, and it may have several clinical consequences. Two of these entities were described in our series.

Eberle et al. [9] consider that tenosynovitis involving the FHL may be the result of repeated friction of the FHL caused by the accessory muscle body of the tendon inside the tarsal tunnel. On the other hand, Wittmayer and Freed [16] suggest that the presence of an accessory muscle body of the FHL should be highly suspected when assessing the MRI scans of a patient with posterior friction syndrome. This anatomical variation has also been identified as a potential causative agent of FHL syndrome associated with a positive passive hallux dorsiflexion test as reported in this series.

Another point to consider is which approach to use when this anatomical variation is identified. Ogut and Ayhan [17] and Eberle et al. [9] have suggested open surgery and a posteromedial approach to resect the accessory fascicle of the FHL. In our series, we did not think resection was necessary, and the clinical results were similar. The accessory FDL may produce tarsal tunnel syndrome, and this concept has been validated by several authors [12, 13, 16, 18–21].

Some authors like Burks and Deheer [18] and Saar and Bell [20] prefer to resect the accessory FDL by means of open surgery and have obtained a good postoperative clinical outcome.

The accessory flexor digitorum longus is an anatomical variation and should be taken into account when performing an arthroscopy of the posterior region of the ankle. By identifying this variation muscle structure lesions will be prevented and we will know how to treat them correctly. However, we have not been able to clearly identify the variation in our cases when we assessed the MRI scans after surgery [22].

The anatomical variation (accessory FDL) described was a finding during an endoscopy of the posterior region of the ankle, and we have spared it by sectioning the superior flexor retinaculum only.

The accessory flexor digitorum longus is an anatomical variation and should be taken into account when performing an arthroscopy of the posterior region of the ankle.

We recommend this treatment on this type of injury although we admit this does not make a definite conclusion.
